# USP18 Mediates Interferon Resistance of Dengue Virus Infection

**DOI:** 10.3389/fmicb.2021.682380

**Published:** 2021-04-30

**Authors:** Haiyan Ye, Xiaoqiong Duan, Min Yao, Lan Kang, Yujia Li, Shilin Li, Bin Li, Limin Chen

**Affiliations:** ^1^Institute of Blood Transfusion, Chinese Academy of Medical Sciences and Peking Union Medical College, Chengdu, China; ^2^Joint – Laboratory of Transfusion-Transmitted Infectious Diseases Between Institute of Blood Transfusion and Nanning Blood Center, Nanning Blood Center, Nanning, China; ^3^Toronto General Research Institute, University of Toronto, Toronto, ON, Canada

**Keywords:** flavivirus, USP18, interferon-α, JAK/STAT signaling pathway, dengue virus

## Abstract

Previous studies demonstrated that dengue virus (DENV) infection developed resistance to type-I interferons (IFNα/β). The underlying mechanism remains unclear. USP18 is a negative regulator of IFNα/β signaling, and its expression level is significantly increased following DENV infection in cell lines and patients’ blood. Our previous study revealed that increased USP18 expression contributed to the IFN-α resistance of Hepatitis C Virus (HCV). However, the role of USP18 in DENV replication and resistance to IFN-α is elusive. In this current study, we aimed to explore the role of USP18 in DENV-2 replication and resistance to IFN-α. The level of USP18 was up-regulated by plasmid transfection and down-regulated by siRNA transfection in Hela cells. USP18, IFN-α, IFN-β expression, and DENV-2 replication were monitored by qRT-PCR and Western blot. The activation of the Jak/STAT signaling pathway was assessed at three levels: p-STAT1/p-STAT2 (Western blot), interferon-stimulated response element (ISRE) activity (Dual-luciferase assay), and interferon-stimulated genes (ISGs) expression (qRT-PCR). Our data showed that DENV-2 infection increased USP18 expression in Hela cells. USP18 overexpression promoted DENV-2 replication, while USP18 silence inhibited DENV-2 replication. Silence of USP18 potentiated the anti-DENV-2 activity of IFN-α through activation of the IFN-α-mediated Jak/STAT signaling pathway as shown by increased expression of p-STAT1/p-STAT2, enhanced ISRE activity, and elevated expression of some ISGs. Our data indicated that USP18 induced by DENV-2 infection is a critical host factor utilized by DENV-2 to confer antagonism on IFN-α.

## Introduction

Dengue virus (DENV), a positive-strand RNA virus, is a member of the arthropod-borne *Flaviviridae* family of viruses; It consists of four serotypes (DENV-1, DENV-2, DENV-3, and DENV-4) and is predominantly transmitted by the bites of infected mosquitoes of the genus *Aedes* (Guzman and Harris, 2015). Infection with DENV is usually asymptomatic but, in some cases, can cause dengue or severe dengue (also known as dengue hemorrhagic fever) (Talarico et al., 2017). It is estimated that DENV infection occurred in over 100 countries and regions, affecting more than 105–390 million people per year (Malavige et al., 2020), which constitutes a major public health concern and poses a substantial social-economic burden. Unfortunately, specific antiviral drugs for treating patients infected with DENV are not available (Dighe et al., 2019), and the mechanisms of pathogenesis during DENV infections are largely unknown.

Innate immunity is the body’s first line of defense to fight against various virus infections. DENV infection triggers innate immune responses through activating the toll-like receptor-3 (TLR3), retinoic acid-inducible gene–I (RIG-I), and melanoma differentiation-associated gene-5 (MDA5) pathways (Nasirudeen et al., 2011; Chazal et al., 2018), Which subsequently induces the host innate immunity, mainly the increased production of type-I interferons (IFN-I, mainly IFN-ɑ, and IFN-β). IFNα/β then binds to the IFN-I receptor (IFNAR) and activates the downstream Jak/STAT signaling pathway to produce a few hundred Interferon Stimulated Genes (ISGs) to limit viral replication/production (Coldbeck-Shackley et al., 2020).

Previous studies demonstrated that IFN-I inhibited DENV replication if the cells were pre-treated with IFN-α before infection, while cells infected with DENV first developed resistance to IFN-α (Diamond et al., 2000; Ho et al., 2005), suggesting that DENV employed some mechanisms to block the anti- DENV effect of IFN-α. Furthermore, DENV can only replicate in mice that lack IFN receptors (Johnson and Roehrig, 1999) or an IFN signaling component, STAT2 (Ashour et al., 2010). These studies indicated that the IFN response is crucial for the host protective immune response to control DENV replication and pathogenesis.

The antiviral effect of IFN-I is exerted by ISGs, some of which are also induced by DENV infection. The role of several ISGs in DENV replication has been reported (Brass et al., 2009; Lin et al., 2009; Jiang et al., 2010; Dai et al., 2011; Helbig et al., 2013; Hishiki et al., 2014; Simon-Loriere et al., 2015; Wang et al., 2018). Ubiquitin-specific protease 18 (USP18) is an ISG, which is rapidly up-regulated by IFN-β treatment by activating the JAK/STAT signaling pathway (Kang et al., 2001). USP18 can also be induced by lipopolysaccharide (LPS) stimulation or virus infection (Li et al., 2016; MacParland et al., 2016). Previous studies have elegantly shown that USP18 is a negative regulator of the IFN-I signaling. Mechanistically, USP18 specifically binds to the IFN-I receptor 2 (IFNAR2) subunit to inhibit Jak/STAT signaling pathway and response to IFN-I (Malakhova et al., 2006).

In our previous studies, we have identified the expression levels of USP18 differed significantly between treatment responders and non-responders to IFN-α-based therapy in patients chronically infected with HCV (Chen et al., 2005). Increased expression of USP18 contributed to IFN-a resistance, while silencing of USP18 potentiated the anti-HCV activity of IFN-α (Randall et al., 2006). Similar findings were reported in pre-treatment liver tissues of patients chronically infected with Hepatitis B virus (HBV) (Xiao et al., 2012), and silencing of USP18 potentiates the anti-HBV activity of IFN-α (Li et al., 2016). Microarray gene expression studies have shown up-regulation of USP18 following DENV infection in HepG2 cells and peripheral blood mononuclear cells (PBMCs) isolated from patients (Fink et al., 2007). However, whether the resistance to IFN-α of DENV correlates with the increased expression of USP18 remains unclear. Both DENV and HCV belong to the *Flaviviridae* family. Therefore, we hypothesized that USP18 might mediate the DENV resistance to IFN-α.

In this current study, we aim to explore the effect of USP18 on the anti-DENV-2 activity of IFN-α *in vitro*. USP18 expression levels were up-regulated by plasmid transfection or inhibited by specific small interference RNA (siRNA), and the effect of USP18 on the replication of DENV-2 was examined in the presence or absence of IFN-α both at mRNA and protein level. The activation of the Jak/STAT signaling pathway following IFN-α stimulation in USP18 knockdown cells was also tested at three levels: p-STAT1/p-STAT2 (Western blot), ISRE activity (Dual-luciferase assay), and ISGs expression (qRT-PCR).

## Materials and Methods

### Cell Culture and DENV Virus

The human cervical cancer cell line (Hela cells) was routinely preserved in our laboratory. Hela cells were cultured in Dulbecco’s modified Eagle’s medium (DMEM) (Hyclone, United States) supplemented with 10% fetal bovine serum (FBS) (PAN Biotech, Germany) and 1% Penicillin-Streptomycin (P/S) (Hyclone, United States) at 37°C in 5% CO_2_ incubator. *Aedes albopictus* cell line (C6/36 cells) and DENV-2 (New Guinea C strain) were generously provided by professor Zhongtian Qi (The Second Military Medical University, China). The cells were cultured in RPMI-1640 (Sangon Biotech, China) supplemented with 10% fetal bovine serum (FBS) (PAN Biotech, Germany) and1% Penicillin-Streptomycin (P/S) (Hyclone, United States) at 28°C in 5% CO_2_ incubator. DENV-2 was amplified and titred in C6/36 cells as previously described (Medina et al., 2012).

### USP18 Plasmid and Transfection

USP18 expression plasmid was constructed with routine molecular cloning techniques. The full-length human USP18 gene was amplified by polymerase chain reaction (PCR) from total RNA isolated from Huh7 cells and cloned into pcDNA3.1-3^∗^tag (Flag, His and StrepII) to create the mammalian expression construct pcDNA3.1-USP18.

Twenty-four hours before transfection, Hela cells were seeded in 6- or 24-wells plate at 2 × 10^5^ cells/mL per well with 2 mL or 500 μL of the complete growth medium, respectively. The transfection mixture was prepared by adding 2 μg plasmid DNA and 4 μg polyethyleneimine (PEI) to 200 μL opt-MEM (Gibco, United States) for 6-wells plated or by mixing 0.5 μg plasmid DNA and 1 μg PEI to 50 μL opt-MEM for a 24-wells plate. The DNA-PEI mixtures were incubated for 15 min at room temperature before adding into each well. Eight hours after transfection, cells were incubated with DENV-2 at a multiplicity of infection (MOI) of 1 for 2 h at 37°C. After that, the cells were washed three times with PBS (Sangon Biotech, China), and a fresh medium was added. Both cells and culture medium from each well were harvested 48 h after infection, and total RNA was prepared for quantitative real-time PCR (qRT-PCR) and cell lysate for Western blot.

### RNA Interference Experiment

USP18 small inhibitory RNA (siUSP18: 5′-CUGCAUAU CUUCUGGUUUATT-3′) and a negative control (Nc) siRNA (NC: 5′-UUCUCCGAACGUGUCACGUTT-3′) were chemically synthesized by Sangon Biotech. Hela cells were seeded in 6- or 24-wells plate at a density of 1 × 10^5^ cells/mL 1 day before transfection. According to the manufacturer’s instructions, the cells were transfected with siUSP18 or Nc at a final concentration of 20 nM using RNAiMAX (Invitrogen, United States). Eight hours after transfection, cells were incubated with DENV-2 at an MOI of 1 for 2 h at 37°C and then cells were washed three times with PBS. Twenty-four hours later, cells were treated with 100 IU/mL IFN-α. Intracellular and extracellular total RNA and protein were extracted 24 h after IFN-α treatment and quantified by qRT-PCR and Western blot.

### RNA Isolation, Reverse Transcription, and Quantitative Real-Time PCR Analysis

Total RNAs in cells and supernatants were extracted by Trizol reagent (Invitrogen, United States) as recommended by manufacturers’ protocols. RNA concentrations were measured by NanoDrop (Thermo, United States), and one microgram of total RNAs was reverse transcribed (Toyobo, Japan) to cDNA, which was amplified in quantitative real-time PCRs with the SYBR Green Realtime Master Mix (Toyobo, Japan) in CFX96 Real-Time PCR System (Bio-Rad, United States). All values were normalized to the level of GAPDH mRNA. Extracellular DENV-2 was quantified by establishing a standard curve as described previously (Hishiki et al., 2014). All the primers used in this study are listed in Table 1.

**TABLE 1:** Real-time PCR primers.

**Gene name**	**Primer sequence**
GAPDH	Forward 5′-GCCTCCTGCACCACCAACTG-3′ Reverse 5′-ACGCCTGCTTCACCACCTTC-3′
Capsid	Forward 5′-CAGATCTCTGATGAATAACCAACG-3′ Reverse 5′-CATTCCAAGTGAGAATCTCTTTGTCA -3′
USP18	Forward 5′-CAGACCCTGACAATCCACCT-3′ Reverse 5′-AGCTCATACTGCCCTCCAGA-3′
IFNα	Forward5′-TCGCCCTTTGCTTTACTGAT-3′ Reverse5′-GGGTCTCAGGGAGATCACAG-3′
IFNβ	Forward 5′-AAACTC ATAGCAGTCTGCA-3′ Reverse 5′-AGGAGATCTTCAGTTTCGGAGG-3′
OAS3	Forward 5′-GTCAAACCCAAGCCACAAGT-3′ Reverse 5′-GGGCGAATGTTCACAAAGTT-3′
Viperin	Forward 5′-TTGGACATTCTCGCTATCTCCT-3′ Reverse 5′-AGTGCTTTGATCTGTTCCGTC-3′
IFIT1	Forward 5′-GCAGCCAAGTTTTACCGAAG-3′ Reverse 5′-GCCCTATCTGGTGATGCAGT-3′

### ISRE-Luciferase Reporter Assay

Hela cells were seeded in a 24-wells plate 1 day before transfection at a density of 1 × 10^5^ cells/mL per well with 500 μL of complete growth medium. The cells were transfected with siUSP18 or Nc at a final concentration of 20 nM for 8 h, and cells were incubated with DENV-2 at an MOI of 1 for 2 h at 37°C. After virus infection, cells were washed three times with PBS. Next, ISRE-Luc reporter plasmid (0.5 μg) plus internal control pRL-TK reporter plasmid (2 ng) were co-transfected into Hela cells. Cells were treated with 100 IU/mL IFN-α (Sangon Biotech, China) for 24 h before harvested for Dual-luciferase reporter assay (Promega, United States).

### Western Blotting Analysis

The cells were harvested and washed three times with PBS and then lysed in radioimmune precipitation assay (RIPA) buffer (Beyotime, China) with PMSF (Biosharp, China). The mixture was centrifuged at 15,000 g for 15 min, and the supernatant was collected. Protein concentration was determined by BCA Protein Assay Kit (Beyotime, China). Twenty five microgram g total protein samples were separated by SDS-polyacrylamide gel electrophoresis and transferred to PVDF membranes (Millipore, United States), and then the membranes were incubated with 5% bovine serum albumin (BSA) with primary antibodies. The primary antibodies used are as follows: rabbit anti-DENV-2 Capsid (Gentex, United States), rabbit anti-STAT1, rabbit anti-p-STAT1 phosphorylated Tyr701, rabbit anti-STAT2, rabbit anti-p-STAT2 phosphorylated Tyr690, rabbit anti-USP18 (Cell Signaling Technology, United States), mouse anti-GAPDH (Zengneng, China). The secondary antibodies included HRP-conjugated ECL goat anti-rabbit IgG or HRP conjugated ECL goat anti-mouse IgG (Beyotime, China). The protein bands were exposed using the ECL Western Blotting Analysis System (Millipore, United States) on ImageQuant LAS 4000 mini (GE, United States). Densitometry was performed with ImageJ software.

### Statistical Analysis

Statistical analyses and calculations were performed with GraphPad Prism 8 software. Student’s *t*-tests were used to determine the difference, and *P*-values less than 0.05 were considered statistically significant. All the data presented are representative of at least 3 independent experiments.

## Results

### USP18 Expression Is Up-Regulated Upon DENV-2 Infection

**FIGURE 1:**
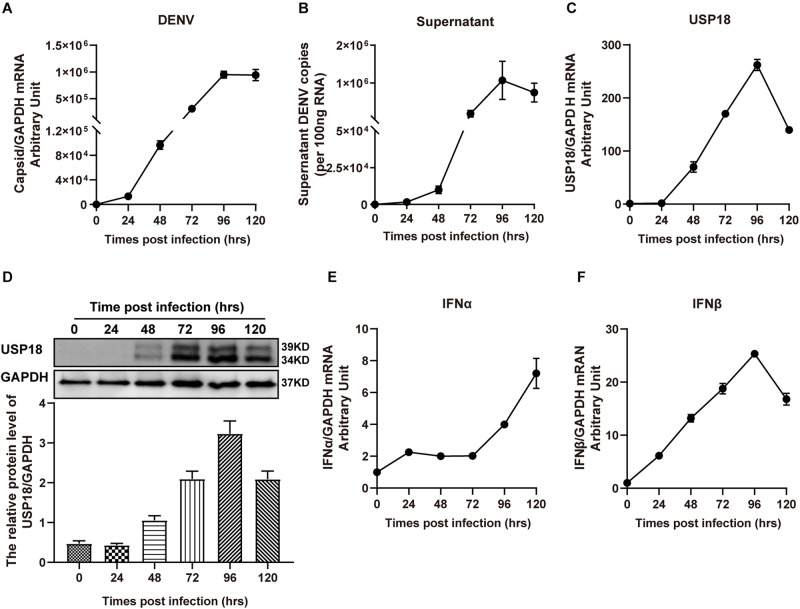
USP18 expression is induced in DENV-2 infected cells. Hela cells and culture medium were collected at various time points post-DENV-2 infection (MOI = 1). Total RNAs were extracted and reverse-transcribed for determining the mRNA levels of DENV-2 RNA **(A,B)**, USP18 **(C)**, IFN-α **(E)**, and IFN-β **(F)** by real-time PCR. Cell lysates were subjected to Western blot analysis of USP18 expression with GAPDH as a loading control, and data were normalized to GAPDH shown as arbitrary units (fold change) **(D)**. Data are presented as mean ± SD. Error bars indicate SD.

Increased USP18 expression has been reported in HepG2 cells and blood samples of patients infected with the dengue virus (Fink et al., 2007). We observed the upregulation of USP18 in DENV-2 infected Hela cells. As shown in Figure 1, DENV-2 RNA levels were increased dramatically in a time-dependent manner in both Hela cells (Figure 1A) and culture supernatant (Figure 1B), indicating the successful infection. In the meantime, USP18 mRNA and protein levels were significantly increased following DENV-2 infection (Figures 1C,D). Next, we want to know how DENV-2 infection-induced USP18 expression. Since clinical data showed that patients infected with DENV during the early febrile period contain high levels of type I interferons in the serum (Kurane et al., 1993; Becquart et al., 2010), we then examined the expression of IFN-α and IFN-β in DENV-2-infected Hela cells. Consistent with the patients’ data, we found IFN-α and IFN-β were both increased significantly following DENV-2 infection, of which the fold change of IFN-β was much greater than that of IFN-α (Figures 1E,F). In addition to Hela cells, we also observed similar results in A549 cells. USP18, IFN-α, and IFN-β were increased following DENV-2 infection (Supplementary Figure 1).

### Over-Expression of USP18 Stimulates While the Silence of USP18 Inhibits DENV-2 Replication

**FIGURE 2:**
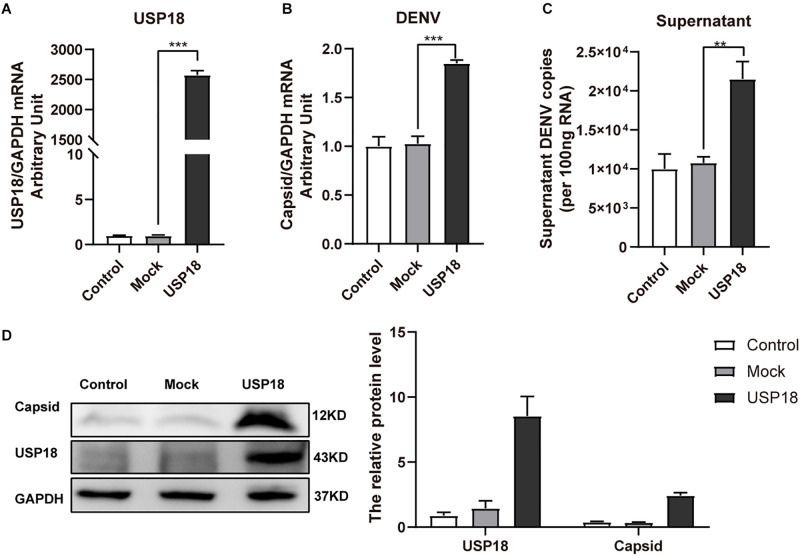
Over-expression of USP18 promotes DENV-2 replication in Hela cells. Hela cells were transfected with USP18 plasmid or empty vector (mock). Eight hours after transfection, cells were infected with DENV-2 (MOI = 1). Cells and culture medium were collected at 48 h post-infection. USP18 mRNA **(A)** and levels of DENV-2 RNA **(B,C)** were determined by real-time PCR. Results were normalized to GAPDH shown as arbitrary units (fold change). Cell lysates were prepared for Western blot analysis of DENV-2 capsid and USP18 expression with GAPDH as a loading control **(D)**. Control is cells infected with DENV-2 only. Data are presented as mean ± SD. Error bars indicate SD; **P < 0.01, ***P < 0.001.

Next, to determine the role of USP18 in DENV replication, we first confirmed whether the USP18 plasmid was successfully constructed. Figure 2A showed that transfection USP18 plasmid led to a remarkable increase of USP18 mRNA expression. We found that USP18 over-expression increased DENV-2 RNA levels in Hela cells and culture supernatants at 48 h post-infection (Figures 2B,C). Western blot further confirmed the over-expression of USP18 and its upregulation effect on DENV-2 capsid protein expression compared to the empty vector (mock) group (Figure 2D). These data indicated that USP18 could stimulate DENV-2 replication within cells and facilitate DENV-2 secretion into the culture medium.

**FIGURE 3:**
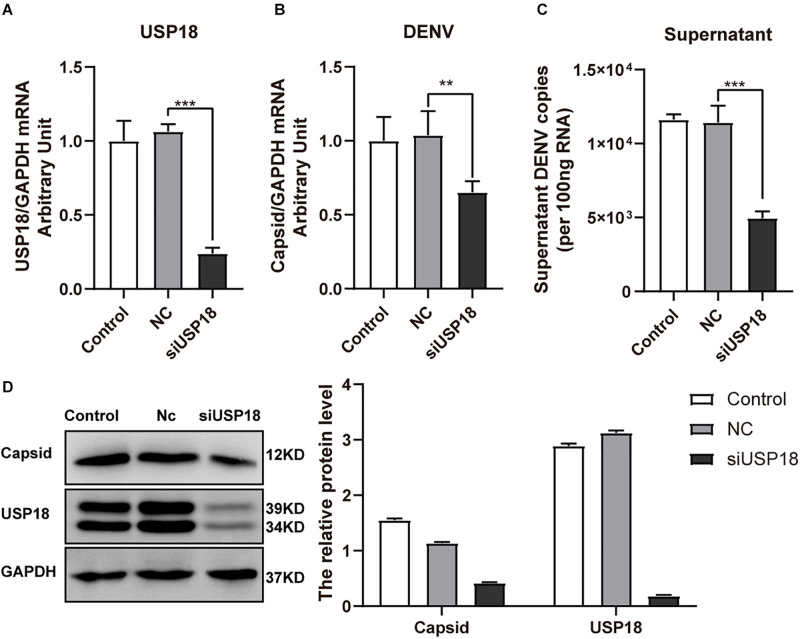
Silencing of USP18 inhibits DENV-2 replication in Hela cells. Hela cells were transfected with siUSP18 or negative control (Nc) for 8 h and then infected with DENV-2 (MOI = 1) for 48 h before cells and culture medium were collected. USP18 knockdown efficiency **(A)** and levels of DENV-2 RNA **(B,C)** were determined by real-time PCR. Results were normalized to GAPDH shown as arbitrary units (fold change). Protein expression of USP18 and DENV-2 capsid was assessed by Western blot with GAPDH as a loading control **(D)**. Control is cells infected with DENV-2 only. Data are presented as mean ± SD; Error bars indicate SD; **P < 0.01, ***P < 0.001.

We also confirmed the effect of USP18 on DENV-2 replication using USP18 siRNA. We found USP18 siRNA could significantly suppress the expression of USP18 mRNA (Figure 3A). And as expected, the intracellular (Figure 3B) and supernatant (Figure 3C) DENV-2 RNA levels were decreased significantly in parallel with USP18 silencing. Western blot confirmed the knockdown of USP18 and its inhibitory effect on DENV-2 capsid protein compared to the negative control (Nc) group (Figure 3D). These results indicated that silencing of USP18 suppressed DENV-2 replication in Hela cells.

### Silencing of USP18 Rescues IFN-α Resistance of DENV-2 Infection

It has been reported that pre-treatment of cells with IFN-α results in the inhibition of DENV-2 replication, while cells developed resistance to IFN-α if they were infected with DENV-2 prior to IFN-α treatment (Diamond et al., 2000; Ho et al., 2005). Since USP18 is a negative regulator of the IFN-I signaling and increased USP18 expression contributed to IFN resistance of HCV (Malakhova et al., 2006; Randall et al., 2006), we then examined whether silencing of USP18 could rescue IFN-α resistance of DENV-2.

**FIGURE 4:**
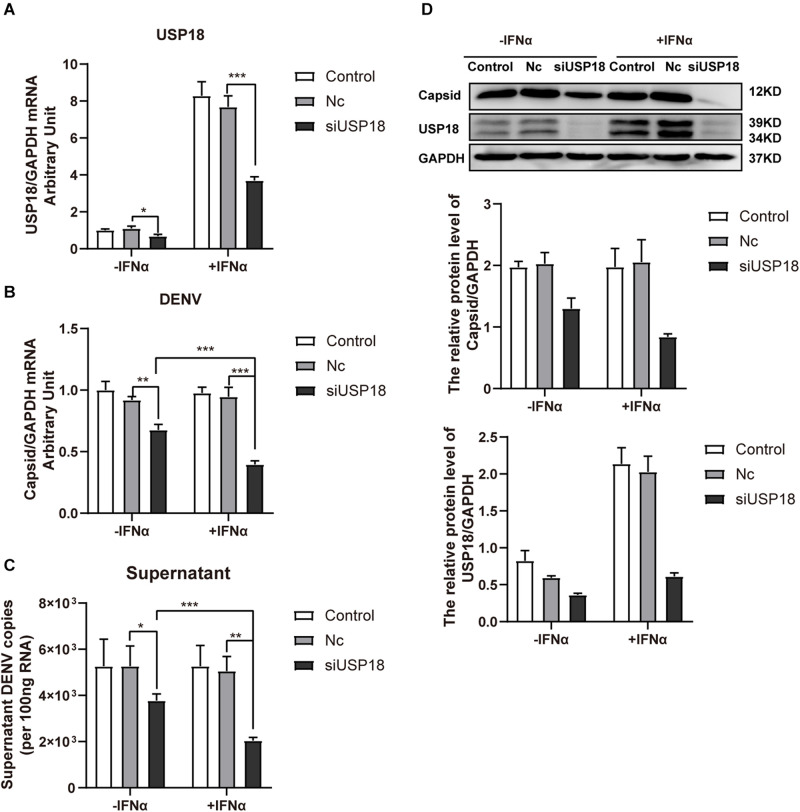
Silencing of USP18 rescues the anti-DENV-2 activity of IFN-α post-DENV infection. Hela cells were transfected with siUSP18 or Nc for 8 h before infected with DENV-2 (MOI = 1) for 24 h, and the cells were treated with 100 IU/mL IFN-α for another 24 h. Cells and culture medium were collected. USP18 knockdown efficiency **(A)** and levels of DENV-2 RNA **(B,C)** were determined by real-time PCR, and results were normalized to GAPDH, yielding arbitrary units (fold change). Protein expression of USP18 and DENV-2 capsid was assessed by Western blot with GAPDH as a loading control **(D)**. –IFNα is without IFN-α treatment, and +IFNα is with IFN-α treatment. Data are presented as mean ± SD, Error bars indicate SD; *P < 0.05, **P < 0.01, ***P < 0.001.

USP18 was significantly knocked down by USP18 siRNA both in the absence and presence of IFN-α than Nc groups (Figure 4A). We found knockdown of USP18 significantly decreased the intracellular (Figure 4B) and supernatant (Figure 4C) DENV-2 RNA levels without IFN-α treatment. IFN-α treatment (100 IU/mL) after infection did not affect DENV-2 replication in Hela cells, consistent with previous reports that post-infection treatment of IFN-α did not influence DENV replication (Diamond et al., 2000; Ho et al., 2005). Knockdown of USP18 rescued the anti-DENV-2 effect of IFN-α and led to decreased DENV-2 RNA in Hela cells (Figure 4B) and supernatant (Figure 4C) compared to IFN-α treatment alone. We also observed DENV-2 RNA levels were significantly decreased in USP18 knockdown Hela cells with IFN-α treatment compared to USP18 knockdown alone (Figures 4B,C). Western blot confirmed the knockdown of USP18 and its inhibitory effect on DENV-2 capsid protein expression in Hela cells (Figure 4D). Collectively, these results suggested that USP18 mediates IFN-α resistance of DENV-2 infection, and silencing of USP18 rescues IFN-α resistance.

### Silencing of USP18 Promotes IFN-α Anti-DENV-2 Activity Through Enhanced Activation of the Jak/STAT Signaling

**FIGURE 5:**
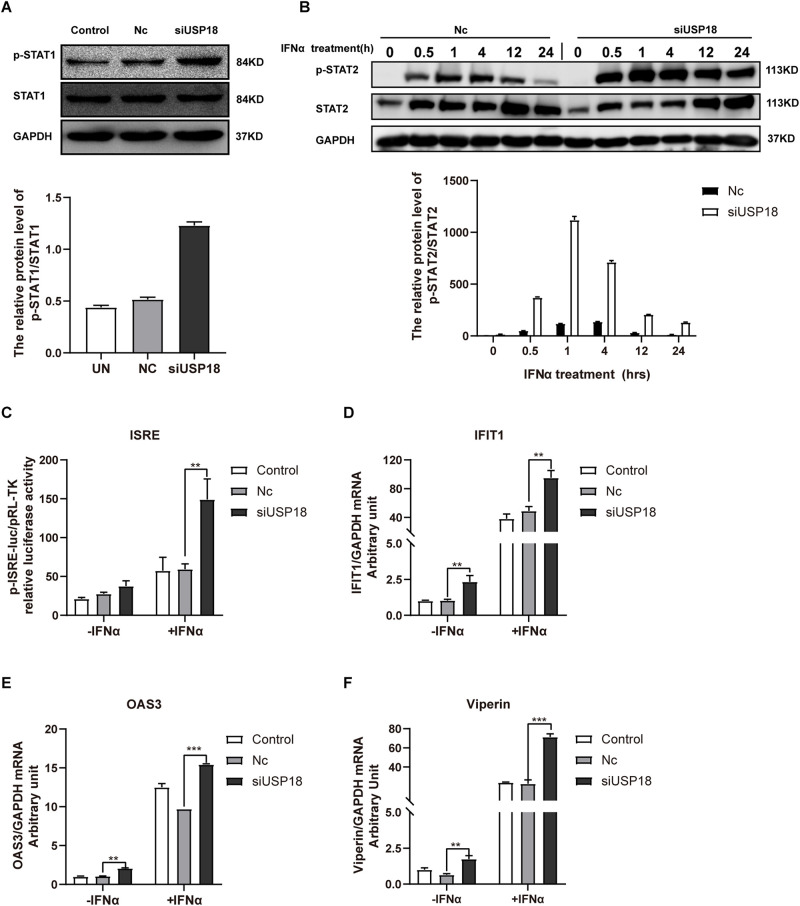
Silencing of USP18 enhanced the activation of the IFN-α-mediated Jak/STAT signaling pathway. Hela cells were transfected with siUSP18 or NC for 8 h before infected with DENV-2 (MOI = 1) for 48 h. The cells were treated with 100 IU/mL IFN-α and harvested 30-min post-treatment. Protein levels of p-STAT1 and STAT1 were analyzed by Western blot with GAPDH as a loading control **(A)**. The cells were treated with 100 IU/mL IFN-α and harvested at 0.5, 1, 4, 12, and 24 h post-treatment. Protein levels of p-STAT2 and STAT2 were analyzed by Western blot with GAPDH as a loading control **(B)**. ISRE activity was determined by Dual-luciferase reporter gene assay **(C)**. Expression levels of IFIT1, OAS3, and Viperin were examined by real-time PCR, and results were normalized to GAPDH, yielding arbitrary units (fold change) **(D–F)**. Control is cells infected with DENV-2 only. –IFNα is without IFN-α treatment, and +IFNα is with IFN-α treatment. Data are presented as mean ± SD; Error bars indicate SD; **P < 0.01, ***P < 0.001.

We then explored the possible mechanism by which silencing USP18 rescued IFNα-resistance of DENV-2 infection. Since the biological activities of IFNs are triggered by the Janus kinase (Jak) signal transducer and activation of transcription (STAT) signaling cascade, we, therefore, examined the activation status of Jak/STAT signaling in USP18-silenced Hela cells infected with DENV-2 in the presence of IFN-α treatment. We observed knockdown of USP18 significantly increased the phosphorylated level of STAT1 (p-STAT1) after 30 min treatment of IFN-α compared with the Nc group (Figure 5A). The phosphorylated level of STAT2 (p-STAT2) increased and prolonged in USP18 knockdown cells after IFN-α treatment compared to the Nc group (Figure 5B). The ISRE activity was enhanced in USP18 knockdown cells compared with the Nc group in the presence of IFN-α (Figure 5C). The mRNA level of some typical ISGs, including IFIT1 (Figure 5D), OAS3 (Figure 5E), and viperin (Figure 5F), were also up-regulated by silencing of USP18 with IFN-α treatment compared with the Nc group. We also observed a significant increase in expression of ISGs (Figures 5D–F) in USP18-knockdown cells in the absence of IFN-α, which may partially explain why silencing USP18 alone inhibited DENV-2 RNA replication without IFN-α treatment. Altogether, these data demonstrated that silencing of USP18 enhanced the activation of the IFN-α-induced Jak/STAT signaling pathway, which may play an important role in the anti-DENV-2 activity of IFN-α.

## Discussion

It has been revealed that USP18 has multiple functions in the regulation of pathological processes, such as pathogen control, cancer development, autoimmune diseases, and neurological disorders (Honke et al., 2016; Kang and Jeon, 2020). USP18 expression is strongly up-regulated in cells stimulated with type I and type III IFNs, polyI:C, lipopolysaccharide (LPS), or tumor necrosis factor-alpha (TNFα) (Kim et al., 2005; Francois-Newton et al., 2011; MacParland et al., 2016). Additionally, USP18 is remarkably induced after viral infection (Li et al., 2016). Previous work reported USP18 expression was up-regulated in both DENV-infected cell lines and patients’ blood samples (Fink et al., 2007). Our present study found DENV-2 infection remarkably induced USP18 expression in Hela cells (Figure 1) and A549 cells (Supplementary Figure 1), consistent with the previous studies. We observed IFNα and IFNβ were significantly increased after DENV infection. Since USP18 is an interferon-stimulated gene, we supposed that USP18 was increased due to IFNα and IFNβ production induced by DENV infection.

USP18 plays a critical role in the innate immune response through at least two independent mechanisms: one is associated with its isopeptidase activity to remove ISG15 from ISG15-conjugated proteins (Malakhov et al., 2002), and the other is to suppress type I IFN signaling through competing with JAK1 for binding to the type I interferon receptor 2 (IFNAR2) subunit and thus blocks IFN-induced Jak/STAT signal transduction (Malakhova et al., 2006). Previous studies have shown that USP18-deficient cells and USP18-knockout mice are hypersensitive to IFN-α treatment (Malakhova et al., 2003). More recent evidence has reported the role of USP18 in innate defense to virus infection (Honke et al., 2016). However, the role of USP18 induced by DENV-2 infection remains unclear. Our study observed overexpression of USP18 significantly increased DENV-2 replication (Figure 2), while knockdown of USP18 decreased DENV-2 replication (Figure 3). Similarly, knockout or knockdown of USP18 in mice or cells exhibit increased antiviral activity against various viruses, including lymphocytic choriomeningitis virus (LCMV), vesicular stomatitis virus (VSV), Sindbis virus (SINV), Hepatitis B virus (HBV), and Human immunodeficiency virus 1 (HIV-1) (Lenschow et al., 2005; Osiak et al., 2005; Kim et al., 2008; Taylor et al., 2018; Li et al., 2020).

Our previous study has demonstrated that USP18 was more highly expressed in the pre-treatment liver tissues of patients chronically infected with HCV who do not respond to subsequent IFN-α treatment (Chen et al., 2005). This result indicated that USP18 might block IFN anti-HCV activity. Indeed, silencing of USP18 potentiated the IFN anti-HCV activity by a fold of 40–100 (Randall et al., 2006). Similar findings were reported in pre-treatment liver tissues of patients chronically infected with HBV (Xiao et al., 2012). Previous work from others and our laboratories showed that USP18 promotes HBV production by inhibiting the type I IFN signaling pathway (Li et al., 2016, 2020).

Interestingly, it has been reported that cells developed IFN resistance following DENV infection. The effect of the IFN-α anti-DENV effect before or after DENV infection differed significantly. Ho et al. concluded that DENV blocked the antiviral effect of IFN-α by observing that viral RNA was actively replicating and viral progeny was abundantly produced in dendritic cells (DCs) infected with DENV, although a large amount of IFN-α was produced (Ho et al., 2001). These data eluded that DENV developed some strategies to desensitize the IFN-α antiviral effect. Following these studies, our study observed that DENV-2 developed IFN-α resistance following infection, although a significant amount of IFN-I was induced (Figure 1 and Supplementary Figure 1). However, elevated and sustained type I IFN response is detrimental, leading to an increase in inflammation (Oon et al., 2016). Therefore, some feedback mechanisms could be required to modulate IFN signaling (Kang and Jeon, 2020), USP18 could act as a key regulator of IFN signaling.

Although IFN-α could exert its anti-DENV-2 activity to suppress DENV-2 RNA replication and production if IFN-α was added before DENV-2 infection, there is little effect of IFN-α on DENV-2 RNA levels when it was added after DENV-2 infection (Figure 4). These results are consistent with data from previous studies (Diamond et al., 2000; Ho et al., 2005), suggesting that DENV-2 antagonizes the antiviral effect of IFN-α. Activating of the Jak/STAT signaling pathway depends on IFN binding to IFNAR. As a negative regulator of the Jak/STAT signaling, USP18 binds to the intracellular domain of IFNAR2 to block the JAK1-IFNAR2 interaction, leading to the inhibition of signal transduction (Malakhova et al., 2006). Therefore, it is reasonable to hypothesize that USP18 is one of the key molecules mediating the IFN-α resistance of DENV-2 infection.

To address the role of USP18 in the IFN-α resistance of DENV-2 infection, we silenced USP18 by specific siRNA before DENV-2 infection and IFN-α treatment. We found that silencing of USP18 rescued IFN-α anti-DENV-2 activity compared to IFN-α treatment alone. We, therefore, concluded that USP18, induced by DENV-2 infection, plays a key role in the IFN-α resistance of DENV-2 infection.

To further explore the mechanism on how silencing of USP18 rescues the IFN-α anti-DENV-2 activity, we examined the activation status of the IFN-α-induced Jak/STAT signaling pathway in USP18-silenced cells in the presence of IFN-α. Data from our study demonstrated that silencing of USP18 enhanced the activation of Jak/STAT signaling as shown by increased expression of p-STAT1 (Figure 5A) and p-STAT2 (Figure 5B), higher activity of ISRE (Figure 5C), and up-regulation of some down-stream ISGs (Figures 5D–F). A few reports have demonstrated that several ISGs played an antiviral role in DENV infection, such as viperin (Helbig et al., 2013), IFIT3 (Hsu et al., 2013), ISG15 (Dai et al., 2011; Hishiki et al., 2014), OAS3 (Lin et al., 2009). However, it is unclear whether USP18 influences the production of interferon during DENV infection. Gu et al. showed that overexpression of duUSP18 (USP18 in ducks) inhibited nuclear factor−κB (NF−κB) and reduced IFN−β production following 5′ppp dsRNA or LPS stimulation (Gu et al., 2019). Previous studies also indicated that USP18 negatively regulated NF−κB signaling to suppress type I IFN production (Yang et al., 2015). Most likely, silencing of USP18 during DENV infection may increase endogenous type I IFN production. Collectively, these data supported our hypothesis that USP18 mediates IFN resistance of DENV-2 infection, and silencing of USP18 could restore the anti-DENV-2 activity of IFN-α.

## Conclusion

In conclusion, we demonstrated that USP18 plays an important role in DENV-2 infection. USP18 expression was induced following DENV-2 infection, and this increased USP18 level blocked the anti-DENV-2 activity of IFN-α. Silencing of USP18 could rescue the IFN resistance of DENV-2 through enhanced activation of the IFN-α-induced Jak/STAT signaling pathway. Accordingly, USP18 might be a good candidate for developing therapeutic agents to control DENV and potentially other viruses’ infections, although the USP18-mediated antiviral mechanism needs to be further investigated.

## Data Availability Statement

The raw data supporting the conclusions of this article will be made available by the authors, without undue reservation.

## Author Contributions

LC, SL, BL, and HY conceived and designed the experiments. HY, MY, and LK performed the experiments. YL and XD analyzed the data. HY drafted the initial manuscript. LC, BL, and XD edited the initial draft. All authors contributed to the article and approved the submitted version.

## Conflict of Interest

The authors declare that the research was conducted in the absence of any commercial or financial relationships that could be construed as a potential conflict of interest.

## Supplementary Material

Supplementary Figure 1 USP18 expression is induced in A549 cells following DENV-2 infection. A549 cells and culture medium were collected at various time points post-DENV-2 infection (MOI = 1). Total RNAs were extracted and reverse-transcribed for determining the mRNA levels of DENV-2 RNA **(A)**, USP18 **(B)**, IFN-α **(C)**, and IFN-β **(D)** by real-time PCR. Data were normalized to GAPDH shown as arbitrary units (fold change). Data are presented as mean ± SD. Error bars indicate SD.
