# Supplementary figures and images for: USP18 Mediates Interferon Resistance of Dengue Virus Infection
Source: Front Microbiol. 2021 Apr 30;12:682380. doi: 10.3389/fmicb.2021.682380 (PMC8130619; doi:10.3389/fmicb.2021.682380)

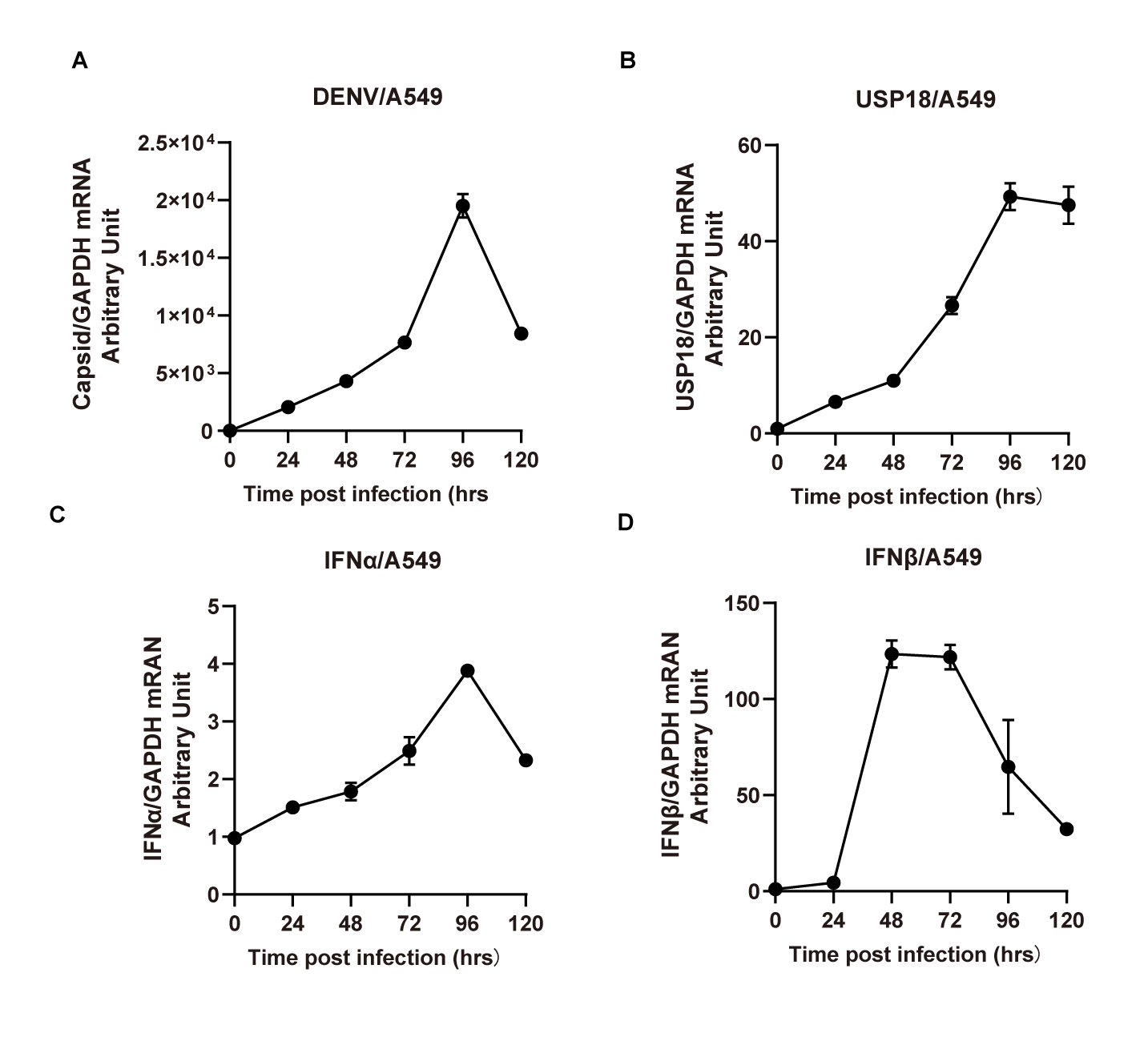

Supplement: Supplementary Figure 1 — USP18 expression is induced in A549 cells following DENV-2 infection. A549 cells and culture medium were collected at various time points post-DENV-2 infection (MOI = 1). Total RNAs were extracted and reverse-transcribed for determining the mRNA levels of DENV-2 RNA (A), USP18 (B), IFN-α (C), and IFN-β (D) by real-time PCR. Data were normalized to GAPDH shown as arbitrary units (fold change). Data are presented as mean ± SD. Error bars indicate SD. [file Image_1.TIF]
